# *Notes from the Field: *Assessment of Rabies Exposure Risk Among Residents of a University Sorority House — Indiana, February 2017

**DOI:** 10.15585/mmwr.mm6705a4

**Published:** 2018-02-09

**Authors:** Betsy Schroeder, Alex Boland, Emily G. Pieracci, Jesse D. Blanton, Brett Peterson, Jennifer Brown

**Affiliations:** ^1^Epidemic Intelligence Service, CDC; ^2^Indiana State Department of Health; ^3^Division of High Consequence Pathogens and Pathology, National Center for Emerging and Zoonotic Infectious Diseases, CDC.

In February 2017, the Indiana State Department of Health (ISDH) was notified of bat exposures at a university sorority house. The initial complaint was made to ISDH because of concerns for food sanitation. Bats had been routinely sighted in shared living areas and hallways. ISDH, in consultation with CDC, collaborated with the university and sorority to assess residents and staff members for potential rabies risk. In 2016, 4.3% of all bats tested in Indiana were positive for rabies. The longest incubation period recorded for indigenously acquired bat rabies is 270 days (*1*); therefore, out of an abundance of caution, ISDH conducted interviews with 140 students and eight employees who resided or worked in the sorority house during the preceding 12 months, all of whom were considered to have possibly been exposed. A web-based survey was administered in February to collect information about bat exposures, which was used to categorize all respondents into having a low, medium, or high risk for rabies exposure per CDC guidance (*2*).

Persons who reported a bite, scratch, or direct skin contact with a bat were categorized as having a high risk. Persons were categorized as having moderate risk if they reported waking and finding a bat in the same room where they were sleeping. Persons who reported no bat exposure were categorized as having a low risk. Respondents categorized as having a high or moderate risk had follow-up interviews in person or by telephone.

Among the 148 possibly exposed persons, 100 (68%) responded to the questionnaire, including 92 (66%) students and all eight employees; 94 respondents reported ever having seen a bat in the sorority house. Among those 94 persons, 70 (74%) reported having seen a bat within the previous 12 months, and 34 (36%) reported seeing a bat ≤1 month ago. Among respondents who reported ever having seen a bat in the sorority house, 13 (14%) were identified as having a moderate or high risk for rabies exposure, including 11 sorority members, one university employee, and one nonsorority member student. After follow-up interviews, nine of these 13 persons were reclassified as having a low risk for rabies exposure. The remaining four persons were considered to have a high (three persons) or a moderate (one) risk. All four persons received a recommendation for postexposure prophylaxis (PEP), which consists of human rabies immune globulin and a series of 4 doses of rabies vaccine. Two persons completed the PEP series during March 20–April 18, and two declined PEP because of a perceived lack of risk. No respondent had developed clinical rabies as of February 2018.

ISDH learned that bats had been roosting in the building for approximately 30 years. Commercial wildlife operators conducted an environmental investigation in March and identified multiple small openings between the house’s exterior wall and doorframe, which can serve as points of ingress or egress for bats. In addition, certain students reported hearing scratching behind a wall inside the house’s common space. This wall was scheduled to be removed as part of a house remodel during summer 2017. A commercial wildlife control operator repaired the openings and completed building remediation during this time. Students returned to the house in August 2017. No bat sightings have been reported since students returned.

This is the first reported instance of a mass bat exposure in a fraternity or sorority house. Multiple high-risk rabies exposures occurred in this sorority house, attributable to bat colonization of the building. The initial complaint to ISDH related to concerns for food sanitation, rather than rabies, is consistent with previous reports indicating an underappreciation of the health risks associated with indoor bat exposures (*3*). ISDH communicated the risk for rabies exposure at meetings with students and university housing directors. All bat exposure events should be reported immediately to public health officials, who can provide advice about rabies risk assessments and determination of the need for PEP.
